# Chitosan-Based Multifunctional Platforms for Local Delivery of Therapeutics

**DOI:** 10.3390/md15030060

**Published:** 2017-03-01

**Authors:** Seong-Chul Hong, Seung-Yup Yoo, Hyeongmin Kim, Jaehwi Lee

**Affiliations:** College of Pharmacy, Chung-Ang University, Seoul 06974, Korea; shotgun30@naver.com (S.-C.H.); marin4906@naver.com (S.-Y.Y.); hm.kim8905@gmail.com (H.K.)

**Keywords:** chitosan, local delivery, anti-cancer drugs, medical device

## Abstract

Chitosan has been widely used as a key biomaterial for the development of drug delivery systems intended to be administered via oral and parenteral routes. In particular, chitosan-based microparticles are the most frequently employed delivery system, along with specialized systems such as hydrogels, nanoparticles and thin films. Based on the progress made in chitosan-based drug delivery systems, the usefulness of chitosan has further expanded to anti-cancer chemoembolization, tissue engineering, and stem cell research. For instance, chitosan has been used to develop embolic materials designed to efficiently occlude the blood vessels by which the oxygen and nutrients are supplied. Indeed, it has been reported to be a promising embolic material. For better anti-cancer effect, embolic materials that can locally release anti-cancer drugs were proposed. In addition, a complex of radioactive materials and chitosan to be locally injected into the liver has been investigated as an efficient therapeutic tool for hepatocellular carcinoma. In line with this, a number of attempts have been explored to use chitosan-based carriers for the delivery of various agents, especially to the site of interest. Thus, in this work, studies where chitosan-based drug delivery systems have successfully been used for local delivery will be presented along with future perspectives.

## 1. Introduction

Oral and intravascular routes are most commonly used for administration of drugs due to their advantages such as patient convenience and fast onset of action. However, drugs administered through these routes are distributed to the whole body via the systemic blood circulation, and thereby only a small portion of the drugs reaches the organs or tissues of interest while most of the drugs are diffused to unwanted areas of the body. This feature of the oral and intravascular administration results in low efficacy of drugs in the intended site and side effects of the drugs in the unwanted tissues [1,2,3]. For this reason, local drug delivery has attracted great attention due to its distinct advantages such as high concentration and efficacy of drugs at the site of interest in the body, decreased side effects of the drugs in the rest of the organs or tissues, and reduced dosing frequency and fluctuation in circulating drug levels [4]. To facilitate local drug delivery, various drug delivery systems of natural or synthetic polymers have been exploited such as particulates at a micro-/nano-scale, hydrogels, and films [5]. The most crucial step for designing such drug delivery systems is to select a polymer with appropriate physico-chemical properties, biocompatibility, biodegradability, and biological characteristics because the polymer largely determines the fate of the local drug delivery systems after administration.

Among diverse biomaterials and synthetic polymers explored as a fundamental material for preparing pharmaceutical formulations for local drug delivery, chitosan, a copolymer consisting of β-(1→4)-linked d-glucosamine and *N*-acetyl-d-glucosamine, has been extensively investigated for the purpose. Chitosan is generally obtained by deacetylation of chitin, which is the primary component of the exoskeleton of many living organisms, in particular marine crustaceans such as shrimps and crabs. By the deacetylation process, chitosan develops primary amine groups in its chemical structure and thereby it is positively charged in diluted acidic aqueous solutions, which makes chitosan distinguishable from other biomaterials [6]. Due to this feature, mechanical properties of chitosan-based drug delivery systems can be controlled by forming a polyelectrolyte complex with other anionic compounds [7,8,9]. In addition, chitosan can be simply fabricated to various morphologies including micro-/nano-spheres, fibers, gels, and films [10,11,12]. The biopolymer can also be conjugated with other molecules through its reactive amino groups of d-glucosamine residues, offering great possibilities to modify the chitosan-based formulations [13,14,15]. Because it is a biomaterial, chitosan exhibits good biocompatibility and biodegradability that can be tuned by varying its molecular weight and deacetylation degree [16,17].

Beyond numerous studies that only focused on investigating the feasibility of chitosan as a main material for fabricating drug delivery systems for simple local drug delivery, innovative chitosan-based platforms with multi-functions have recently been emerging. With a variety of approaches for conferring different functions of chitosan, the chitosan-based drug delivery systems have demonstrated versatile properties, thereby overcoming major challenges posed in diverse research fields. In particular, in the field of cancer therapy and tissue regeneration, much literature has recently reported remarkable achievements using the chitosan-based multifunctional systems (Table 1). For further studies to advance the chitosan-based systems, deep understanding of approaches for designing the drug delivery systems of chitosan with different functions that have been done to date is indispensable. Thus, in this review, representative chitosan-based multifunctional platforms for local drug delivery will be introduced, mainly focusing on their applications for cancer therapy and tissue regeneration.

## 2. Conventional Chitosan-Based Local Drug Delivery

### 2.1. Mucosal Surface

**Figure 1:**
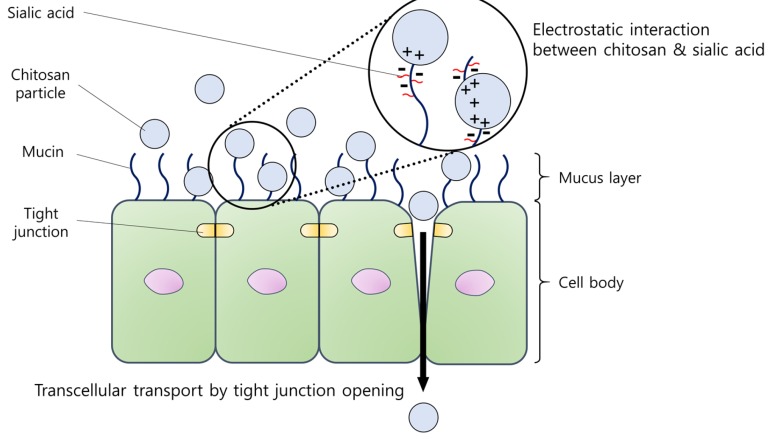
A diagrammatic presentation of the electrostatic interaction between chitosan particles and the mucus layer. Positively charged chitosan particles are prone to interact with the negatively charged sialic acid, resulting in enhanced adherence. In addition, a tight junction opening could be induced by chitosan particles, promoting local delivery of drugs.

Most human organs are covered by a slippery material, namely mucus. The mucus is mainly composed of glycoproteins called mucins, in which a large portion of sialic acid residues take place. Due to the abundant sialic acid residues, the mucus layer shows a negatively charged surface [18], which provides a favorable environment for chitosan to be electronically adhered (Figure 1). Many researchers have used such a phenomenon to deliver the drug locally to the mucus-covered organs [19,20,21].

To effectively deliver drugs into the eye, one must overcome lacrimal elimination in order to provide sufficient time for drugs to penetrate [22]. Pavan et al. [23] produced ultra-small chitosan nanoparticles (US-CNPs) for the encapsulation of bovine lactoferrin to treat pesticide-induced ocular toxicity. The synthesized CNPs were small in size, ranging about 30–50 nm. The biodistribution results demonstrated that a large portion of US-CNPs were presented on the mucus layer compared to large CNPs or poly(lactide-*co*-glycolide) (PLGA)-based NPs. In addition, they were able to penetrate into the rat eye even at low concentration (100 μg/mL) compared to conventional CNPs. The interaction between chitosan and the mucus layer resulted in prolonged contact time on the eye, which might have assisted the penetration [24].

Researchers are interested in the fact that nasal administration is an effective delivery route to the central nervous system. It is found that direct diffusion of drugs through olfactory epithelium allows to circumvent the blood–brain barrier [38]. Matthias et al. [39] developed siRNA conjugated CNPs for nose-to-brain delivery to treat glioblastoma. The electric interaction of positively charged chitosan and negatively charged siRNAs successfully encapsulated siRNAs in the nanoparticles and protected them from RNases. The administered CNPs strongly adhered to the nasal mucosa and the siRNAs were detectable up to 8 h after administration, compared to naked siRNAs which showed only mild adherence. In addition, encapsulated siRNAs were effectively transported to glioma cells through nasal cavity. These results were the outcome of the combined effect of chitosan. The mucoadhesive property of chitosan allowed the CNPs to overcome mucosal clearance in the nasal cavity. Afterwards, tight junction opening of the polymer [40] made it easier to transport CNPs through olfactory epithelium. These features make CNPs a promising nose-to-brain local drug delivery system.

Local drug delivery to the oral cavity using chitosan was mainly studied to treat oral candidiasis. To treat such disease, Seda et al. [41] developed chitosan-coated nanoparticles (CS-NPs) containing fluconazole (FLZ) for local treatment. The team performed an in vitro release study as well as an ex vivo diffusion test, and in vivo anti-fungal observations using rabbits as the animal model. Not surprisingly, the CS-NPs were able to interact with mucins, prolong the release of the drug, and successful recovery from candidiasis was seen in rabbit oral cavities. An interesting result from the ex vivo Franz diffusion cell test using cow buccal mucosa was observed. No drug was found from the receptor phase samples, but rather 20% of FLZ was found in the mucosa. This indicates that CS-NPs resided locally to reach effective salivary concentration of FLZ, and did not penetrate through the buccal membrane for systemic absorption.

### 2.2. Skin Surface

In dermal delivery, the use of chitosan exhibits many benefits. Even though the outermost skin layer is not covered with mucus, the slight negative charge of mammalian skin offers a suitable environment for chitosan to be adhered [42]. Interestingly, it was studied that positively charged particles are less prone to deeper skin penetration, thus showing less systemic exposure, which is also another benefit of using chitosan for local delivery [43].

In recent years, lecithin-chitosan nanoparticles (LC-CNPs) have been largely studied for dermal delivery. Lecithin, a natural lipid mixture of phospholipids, has negative charge which makes it prone to interact with positively charged chitosan [44]. Qi et al. [45] studied LC-CNPs as the dermal delivery system for quercetin, an anti-oxidant. Both in vitro and in vivo study of permeation studies showed significant drug accumulation, especially in the epidermis, compared to quercetin propylene glycol solution. Ipek et al. [46] and Taner et al. [47] produced LC-CNPs and further incorporated them into chitosan gel for more suitable formulation. These particles are both in agreement that the chitosan gel formulation had fair compatibility with skin pH, sufficient rheological and mechanical properties for topical administration. In addition, when these LC-CNPs were incorporated into chitosan gel, they showed better efficacy compared to commercial cream, even though the drug amount of LC-CNPs was 10 times less than the latter. Again, the LC-CNPs of the two particles also showed higher drug accumulation in the epidermis. The fact that LC-CNPs did not penetrate further into deep dermis is in alignment with the benefit of positively charged chitosan, again emphasizing the advantage of using chitosan for the skin delivery system.

## 3. Diversity of Chitosan-Based Local Drug Delivery

### 3.1. Cancer Therapy

#### 3.1.1. Chitosan-Based Platforms for Embolic Therapy

**Figure 2:**
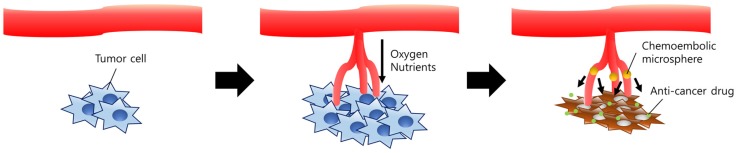
Schematic diagram of the therapeutic mechanism of chemoembolic microspheres. Tumor cells rapidly proliferate due to the angiogenesis. Oxygen and nutrients are supplied via newly generated arteries. Chemoembolic microspheres efficiently occlude vessels which were formed by angiogenesis, leading to starvation of the tumor tissue. Drugs released from the microspheres exhibit a synergistic anti-cancer effect on the tumor tissue.

Anti-cancer drugs for chemotherapy have been continually studied for decades. Although they are largely developed up to date, some fundamental problems of anti-cancer drugs are still being mentioned. The main drawback of these drugs is severe side effects due to their systemic administration and low specificity to the tumor tissues [48]. Thus, there have been unmet needs for targeted local delivery. Selective embolization is an attractive therapy to these needs because it uses a typical feature of cancer cells, angiogenesis [49]. By occluding arteries generated by cancer cells, embolic materials can induce starvation of cancer cells (Figure 2). Until now, many types of embolic materials, such as polyvinyl alcohol (PVA) [50] or PLGA [51] were used. Despite its potency of cheapness as well as biocompatibility, non-toxicity, and biodegradability [52], chitosan has been disregarded as a candidate due to its brittleness when formulated into microspheres.

In this background, Kang et al. [25] focused on preparing deformable chitosan microspheres (CMs) for embolization by ionotropic gelation with polyethylene glycol (PEG). PEG was used as a porogen and was extracted later to form porous CMs, which made them overcome their rigidity. Stable pore structures were observed in SEM analysis and water retention increased proportionally with the amount of PEG. Although blended shapes were observed when passing through the narrow end of the catheter, they recovered their original form after administration. These results indicated that porous CMs could be a great candidate for embolization material which would be able to overcome clogging problems in catheters [53]. A later study continued by Park et al. [27] investigated CMs as a multifunctional embolic platform. Park and his team formulated doxorubicin (DX)-loaded CMs to present the synergistic effect of embolization and sustained-release of the anti-cancer drug, that is, chemoembolization. Tripolyphosphates were used to load DX more efficiently by neutralizing the positive charge of chitosan and to enhance the sustained-release profile. The DX-loaded CMs were spherical in shape, deformable, and homogeneous in size, satisfying the essential factors of efficient chemoembolization [54]. An in vitro drug release test showed an initial burst, followed by sustained release of DX. These features resulted in an efficient anti-cancer effect in the in vivo test. Tumor size was decreased by about one-third of the initial size in CMs treated groups, whereas other groups treated with normal saline, DX solution, and blank chitosan microspheres showed increased tumor size. Kim et al. [55] conducted another study to improve conventional CMs for chemoembolization. They noticed that the drug loading prior to clinical use was time consuming and the drug release was instable. The team planned to overcome these defects by loading drug encapsulated liposome in CMs. It was found that liposomal CMs presented a stable sustained-release profile of DX, while liposomal DX without CMs showed a burst release of DX. Combining with previous studies, these results indicate that liposomal CMs could be used for sustained-release chemoembolization.

Another consideration of CMs for embolization is monitoring them. Although CMs can be delivered selectively to tumor arteries, the help of X-ray angiography is almost inevitable [56]. Since excessive exposure to X-rays is harmful [57], patients would be safer if physicians could observe their changes visually with reduced usage of them. Kang et al. [58] used superparamagnetic iron oxide nanoparticles (SPIOs), nanocrystals of iron oxides coated with hydrophilic polymers, to achieve such an objective. SPIOs are originally used as a contrast agent for magnetic resonance imaging (MRI). Therefore, they hypothesized that SPIOs loaded CMs could be traceable via MRI. Spherical CMs with narrow size distribution were prepared by emulsion and the cross-linking method. The amount of loaded SPIOs was larger than the detectable amount of an early report [59], indicating possibility for detectability. It was also revealed that highly-cross linked CMs had a lower degree of swelling, leading to secure entrapment of SPIOs. The study was continued by Chung et al. [28] to strengthen the feasibility for detectable embolization. SPIO-CMs were prepared by ionotropic gelation including pores that made them deformable. SPIOs were loaded with minimal loss, showing 94% of loading efficiency. The microspheres passed through the catheter without any damage, confirming their deformability. In vivo MR tracing in renal arteries of rabbit showed long-term occlusion of target blood vessels for 18 weeks. The effect of embolization was further supported by anatomical observation and Prussian blue staining. The embolized kidney showed decreased size 8 weeks after treatment. Prussian blue staining indicated SPIO-CMs located in the main and segmental renal arteries, enhancing evidence of selective embolization.

#### 3.1.2. Chitosan-Based Platforms for Theragnosis

Diagnosis is an important aspect for cancer therapy, because accurate diagnosis can give information regarding whether a treatment will be effective to the individual patient [60]. Molecular imaging is an evolving area of diagnosis due to its non-invasive feature and real-time monitoring [61]. Thus, molecular imaging was clinically used with diverse imaging modalities, by loading contrast agents to the molecular carrier. Nanoparticles have been the focus for clinical diagnosis of cancer via molecular imaging since they can be efficiently accumulated to tumor tissues by the enhanced permeation and retention (EPR) effect [62]. Besides, there have been numerous studies to encapsulate anti-cancer drugs into nanoparticles for cancer therapy. By integrating these two strategies, a new concept called ‘theragnosis’ has come to the fore. The concept of theragnosis is attractive because of its possibility of personalized therapy [63]. Chitosan is a bio-friendly material that is ideal for nanoparticle-based theragnosis, since it can be easily formulated into nanoparticles making it suitable for the EPR effect [64,65]. It is also degradable in human bodies after its diagnostic and drug delivering role. Kim et al. [30] investigated the feasibility of chitosan nanoparticles (CNPs) for theragnosis. CNPs were formulated to encapsulate paclitaxel (PTX) and conjugated with Cy5.5, a near-infrared fluorescent (NIRF) dye. When intravenously injected, CNPs were traceable in real-time by in vivo NIRF imaging, showing a tumor-selective signal proportional to tumor size. The therapeutic effect also could be observed by NIRF imaging. Tumor growth rates were investigated and CNPs proved their feasibility for cancer therapy, showing reduced tumor volume compared to normal saline groups.

Theragnosis can be achieved not only by optical imaging but also by other techniques such as MRIs. An additional study conducted by Lim et al. [31] traced the CNPs by MRI. The research team also attempted to enhance the selectivity of CNPs. They focused on a feature of cancer cells that exhibit abnormally high acidic environment, which makes them distinguishable from normal cells [66]. Thus, pH-sensitive formulation was suggested to be a potent drug delivery system for cancer therapy. pH-sensitive CNPs were prepared by adding maleoyl groups on the chitosan backbone. Moreover, magnetic nanocrystals (MNCs) were encapsulated into CNPs, making them detectable by MRI. CNPs showed increased particle size in pH 5.5 due to the hydrolysis of maleoyl groups. In accordance with hydrolysis, 90% of DX was released within 24 h, while only 20% did at higher pH conditions (pH 7.4 and 9.8). When intravenously injected in mice, MR contrast effects were seen in tumor tissues by the EPR effect and 80% of DX was released from CNPs because of acidic condition, as expected. CNPs also reduced the tumor growth rate, compared to naive DX and normal saline.

Although diverse imaging techniques for diagnosis have been used clinically, limitations of conventional techniques still act as an obstacle for accurate diagnosis. Positron emission tomography (PET) is a good example. PET has the advantage that it can provide long-term image and quantitative information [67,68]. Utilizing these advantages, PET is applied to diagnose many types of cancer such as lung, colorectal, and breast cancers [69,70,71]. However, there is a limitation; it was difficult to detect specific molecular activity with PET, reported by Lee et al. [72]. To overcome this defect, Lee and his colleagues investigated the application of various imaging techniques in one formulation simultaneously. CNPs were radiolabeled with Cu^64^ for PET imaging and conjugated with special moieties which can be activated by matrix metalloproteinase (MMP) for NIRF imaging. Since MMPs are overexpressed in tumor cells [73,74], they could be molecular targets for theragnosis. Results from NIRF imaging showed rapid response, showing a plateau 6 h after intravenous injection. In contrast, PET images presented their peak response 24 h after injection and it was maintained over 48 h. Both images showed accumulation of CNPs in tumor tissues. Multimodal CNPs showed integrated advantages of two methods; quantitative information and molecular accuracy. These results indicate that multimodal CNPs could be promising theranosis agents.

#### 3.1.3. Chitosan-Based Platforms for Cancer Radiotherapy

Radiotherapy is a concept using radiation for therapeutic purposes. Beta-emitting materials have been considered as useful anti-cancer therapeutics because of their cytotoxic activity [75]. Although radiotherapy was expected as a novel therapy for cancers, there were also disadvantages that limit their clinical usage. Kim et al. [76] emphasized safety issues. They claimed that because the distribution of the radioisotope is dependent on tumor vasculature, leakage can occur if vascular-disturbing incidents happen. Therefore, the need for local isotope delivery has risen and studies about the biodistribution of the isotope were conducted. A biodistribution study of the holmium-166–chitosan complex conducted by Yuka et al. indicated that it could be a potential candidate for tumor radiotherapy [77]. 166-Holmium (^166^Ho) possesses an appropriate half-life of 26.8 h and a high beta energy of 1.85 MeV which make it suitable for the therapeutic radioisotope. Meanwhile, chitosan has the physicochemical feature that it is soluble in acidic conditions but insoluble in neutral or basic conditions. In their reports, it was also suggested that chitosan, a biocompatible material, can form chelate with ^166^Ho. It means that acidic solutions can be prepared for stable ^166^Ho–chitosan injection. Once injected into human organs, chitosan forms a solid structure, establishing a radioactive pharmaceutical device. In the results, the radioactivity of ^166^Ho–chitosan was localized in the administration site when intrahepatically and intratumorally injected, while formulation with ^166^Ho alone spread to the whole body via the vascular system. A step further, Lee et al. [78] researched the feasibility of the ^166^Ho–chitosan complex as a therapeutic agent for hepatocellular carcinoma. The ^166^Ho–chitosan complex showed coagulation necrosis in vivo because of the radiation effect of ^166^Ho, when injected directly to tumor. The area of necrosis was proportional to the dosage and the required dosage for total necrosis was dependent on the size of the tumor. It was also found that ^166^Ho–chitosan accumulated within the tumor, showing no significant leakage in neighboring organs. Furthermore, ^166^Ho–chitosan formulation for hepatocarcinoma successfully finished its phase IIb clinical trial [76]. Forty patients participated in this clinical trial and they were treated with ^166^Ho–chitosan formulation which is designed to be injected intratumorally. A total of 77.5% of patients experienced complete tumor necrosis. The ^166^Ho-chitsoan complex proved its effect clinically and later, was approved by Korean Food and Drug Administration (KFDA) for hepatocarninoma (Milican, DongWha Pharmaceutical Co., Seoul, Korea).

### 3.2. Tissue Regeneration

#### 3.2.1. Chitosan-Based Platforms for Enhanced Cell Adhesion Properties

**Figure 3:**
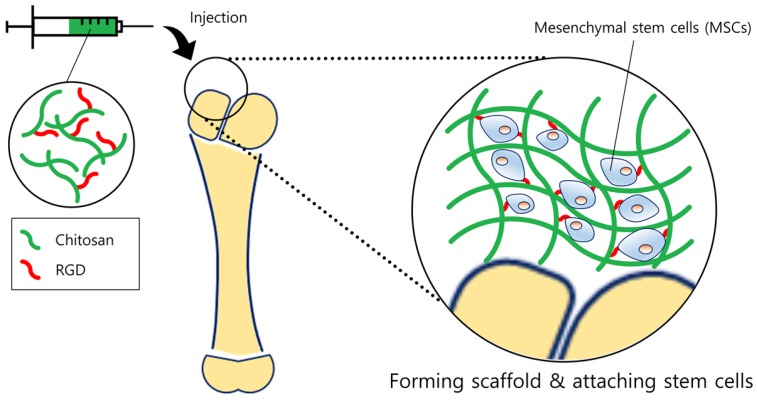
Schematic diagram of tissue regeneration. Chitosan forms a scaffold similar to extracellular matrix (ECMs), providing a suitable environment for cells to be adhered. In order to enhance the attachment of stem cells, RGD moieties were linked with the chitosan backbone. Stem cells then could proliferate on the scaffold more efficiently, ultimately regenerating the bone or cartilage tissue.

Tissue engineering is a promising method for tissue regeneration. By providing an artificial extracellular matrix (ECM) platform, cells can adhere and grow in the platform. The non-immunogenicity, low allergenicity, biodegradability, formability, and chemical variability of chitosan allow us to produce various types of platforms for diverse tissue engineering. Injectable hydrogels have been widely used as scaffolds and have several advantages for tissue engineering; minimal invasive procedure, physical flexibility, and easiness of incorporating cells. However, one critical limitation is the lack of adhesion in some cells to scaffolds. It is necessary to increase the cell adhesion rate in order to successfully load the desired cell and regenerate the tissue. Dhanya et al. [33] made an attempt to prepare a scaffold for adipose tissue engineering by improving conventional alginate hydrogels. Although alginate hydrogels have been widely used as a biomaterial for tissue regeneration, lack of cell adhesion was the limitation for its clinical usage. *O*-carboxymethyl chitosan (O-CMC) was adopted to solve this problem, due to its good water solubility, high viscosity, and high hydrodynamic volume. The alginate/O-CMC/nano-fibrin (AOF) scaffold was found to have high viscous moduli when measured by the rheometer, proving it as a useful scaffold for cell incorporation. In addition, AOF showed a relatively rapid and higher swelling ratio than PVA due to abundant hydrophilic groups which can help the scaffold to be hydrated. This demonstrates that the AOF can obtain a sufficient area/volume ratio and porosity which allows cells to be infiltrated into scaffolds more efficiently. Coinciding with the results, adipose-derived stem cells were well attached, proliferated, and differentiated in culture. To increase cell adhesion, another useful method is to conjugate the cell-binding sequence arginine-glycine-aspartate (RGD) to chitosan. However, one of the drawbacks of using RGD-chitosan is the difficulty of conjugating RGD uniformly and simultaneously crosslinking the scaffold. To overcome this drawback, Tsai and his team previously developed a specific method to fabricate a RGD-conjugated, crosslinked chitosan scaffold for bone tissue engineering (Figure 3), described elsewhere [79]. Using their method, the team evaluated the suitability of RGD-chitosan scaffolds for bone tissue engineering using mesenchymal stem cells (MSCs) [34]. By visually observing the attachment of MSCs, RGD-chitosan film displayed elongated and spread morphology compared to normal chitosan film, on which only few cells were visible. Further quantification of cell numbers also confirmed that the number of attached MSCs on RGD-chitosan film was approximately twice the number observed on normal chitosan. This result is in alignment with RGD-chitosan scaffold results, which also showed a significant increase in MSCs attachment. Moreover, MSCs seeded in the RGD-chitosan scaffold were able to successfully differentiate, showing the highest alkaline phosphatase activity and calcium deposition. These results indicate that RGD-chitosan scaffolds can thus act as a favorable environment for the MSCs to adhere.

A chitosan-based platform can also be used in a more delicate area. One of the good examples is a cardiac patch for a congenital heart defect, a defect in the contractile myocardial tissue. Although there are surgical patches on the market to treat this type of defects, there have been problems such as inabilities to grow and regenerate cells, and limited durability. Incorporating stem cells into the chitosan scaffold can be a solution to these drawbacks. Pok et al. [80] prepared a gelatin-chitosan hydrogel-based multilayer cardiac patch. They adopted polycaprolactone (PCL) to attain high patch tensile strength, and gelatin to consolidate cell attachments. Chitosan was the main component of the patch, because it is biodegradable and can be fabricated into a porous structure for cell migration easily by freeze-drying the gel solutions. Multilayer scaffolds—50:50 (gelatin:chitosan)—showed a narrow pore size distribution and moderate porosity, suggesting an effective mixture ratio. Samples with such a ratio demonstrated their potential by presenting high cell attachment and viability.

#### 3.2.2. Chitosan-Based Platforms for Enhanced Wound Healing

**Figure 4:**
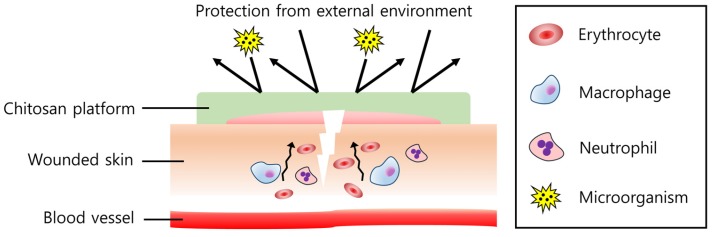
Effect of the chitosan platform on wound healing. Chitosan attracts immune cells such as macrophages and neutrophils, regulating inflammatory responses to recover the wound quickly. Erythrocytes and platelets (not described in the figure) also agglomerate at the wound site, showing hemostasis. In addition, a harmful external environment and microorganisms can be efficiently isolated by the chitosan platform.

Wound healing products are another medical platform of chitosan. Chitosan was found to influence all steps of wound healing [81] by regulating the immune system. It can attract neutrophils and macrophages towards the wound, controlling fibroplasia and reepithelization [82] (Figure 4). Anti-microbial and hemostatic effects also make chitosan a potential wound healing material [83]. In addition, chitosan has biocompatibility, biodegradability, bioadhesiveness, and low toxicity, which makes it more a powerful candidate for wound healing. Therefore, by combining chitosan with other materials which assist the healing process, the multi-functional effect could be obtained, thereby having a synergistic effect in wound healing.

In this background, Lee et al. [36] investigated the hemostatic and vascular healing effect of chitosan film in combination with epidermal growth factor (EGF). Compared to a formulation on the market, both single chitosan film and EGF-chitosan film showed similar in vivo hemostatic activity. However, in a histological study, EGF-chitosan film showed a significantly improved vascular healing effect. The researchers proposed that impregnating EGF to chitosan film does not interrupt the action of each other, but rather they have a synergistic effect of vascular healing and hemostasis. Proving its effectiveness, applications of chitosan have been extended to treat diverse types of wounds such as diabetic wounds [37]. Moura et al. fabricated foams with chitosan derivatives to treat diabetic wounds. Chitosan foams were prepared using a chitosan derivative, 5-methyl pyrrolidinone chitosan (MPC), to cover the wound site and help regeneration. A bioactive neuropeptide neurotensin (NT) [84] was added in order to improve wound healing. Significantly decreasing the wound size, the result suggested a major wound healing impact of NT-loaded MPC foams in diabetic animals, not to mention the sustained release of NT due to MPC. In addition, the combined formulation could modulate the immune response, which is also important in the healing process.

### 3.3. Chitosan-Based Platforms for Miscellaneous Therapeutic Purposes

Chitosan, in combination with various materials—newly attempted multiplatforms—has been developed to improve therapeutic effects. Recently, Behl et al. [85] developed a contact lens containing chitosan nanoparticles (CNPs) as an alternative to eye drops. The negatively charged dexamethasone sodium phosphate (DXP) could be successfully incorporated into positively charged chitosan by ionic interaction to form stable chitosan nanoparticles (DXP-CNPs). These nanoparticles were then mixed with polymer material and molded to form a small lens. The CNPs mixed lens was found to have an average transmittance of 95%–98%, with almost no visual disturbance and no irritation to the eye expected, giving the advantage of using chitosan in combination with such formulation. The mucoadhesive CNPs can thus adhere to the mucus layer of the eye, offering prolonged residence time and resistance to lacrimal elimination [86]. Also, the release rate of DXP from the lens was nearly three-fold slower than the DXP-CNPs itself, demonstrating the combined effect of the lens which acts as an additional obstacle that slows down the drug release [87]. The authors calculated that compared to conventional DXP eye drops, an increase of up to 72% in bioavailability could be reached with this formulation. Another formulation, such as a hydrogel, can also be useful to increase the retention time and bioavailability of the drug. Cho et al. [88] previously developed a glycol chitosan-based thermogel for biomedical applications. The authors chemically cross-linked hexanoyl glycol chitosan (HGC) by exposing it to UV light. In this study, enhanced thermosensitivity and low sol-gel transition temperature of the polymer was observed even at low concentrations (3–5 wt. %) of chitosan compared to typical thermogelling polymers [89]. Again, a more recent study by Cho et al. [90] created a HGC-based thermosensitive gel with the combination of the anti-glaucoma drug brimonide tartarate (BRT). The BRT-HGC showed a promising result in which the viscosity was increased at body temperature and prolonged intraocular pressure decrease was observed, compared to the conventional eye drop. The advantage of the low concentration requirement of chitosan along with body temperature-activated rheology proved chitosan as the cost-effective polymer for its use. As a new multiplatform for dermal use, Tu et al. [91] combined the use of *N*-trimethyl chitosan nanoparticles (TMCNPs) with polypropylene electret to promote the dermal delivery of protein drugs. Electret is a dielectric material that can hold onto its electric charge for a long time. The research team hypothesized and verified the fact that with the use of both positively charged electret and TMCNPs, improved penetration of TMCNPs into the epidermis and dermis could be observed. They proposed a powerful electrostatic repulsive force between TMCNPs and electret as the mechanism for successful dermal delivery. In addition, the tight junction opening of chitosan also acts as a delivery enhancing effect [92]. If the degree of repulsive force could be adjusted just enough to deliver CNPs only to the upper skin, this combined formulation could be a promising method for effectively delivering drugs without systemic absorption.

## 4. Conclusions and Future Perspectives

Under the increasing demand of local drug delivery, amongst various polymers, chitosan, a bio-friendly and easily obtainable polymer, has been widely exploited for the purpose. Chitosan with its derivatives plays a predominant role in medical and pharmaceutical sciences. Its biocompatibility, biodegradability, and non-toxicity promote the use of chitosan as a useful drug carrier to the human body. As documented in the previous sections, chitosan also has unique properties that coincide with its specific use. For example, the mucoadhesive property of chitosan benefits the targeted delivery to the mucus-covered organs and induces prolonged residence time of drugs. The tight junction opening property of the polymer allows the drugs to penetrate more easily through the organs, thereby increasing the accumulation of the drugs at the target of interest. In addition, the chemical structure of chitosan is easily modified due to the reactive primary amines on the chitosan backbone. To sum up, these unique properties have given chitosan the privilege of being frequently used as a fundamental material for fabricating local drug delivery systems.

Innovative chitosan-based multifunctional platforms are continuously under development as versatile characteristics of chitosan are being understood. However, there are still some unsolved problems that must be taken into account when applying chitosan formulations. When chitosan is combined with other polymers or synthesized into diverse derivatives, the safety issue must be considered as the principal factor. Many more chitosan mixtures and derivatives are being explored to develop novel multifunctional platforms for local drug delivery. In response to such drastic development, the lack of toxicity studies is the primary assignment that needs to be fulfilled. The majority of toxicity studies on the platforms are limited to in vitro tests. Thus, performing additional in vivo or ex vivo studies will help to ensure its safety. Furthermore, clinical studies on human bodies should be carefully conducted based on these results. Along with its safety, the stability of the platform should also be taken into account. Parameters such as degradation time or elimination rate should be carefully controlled in order to validate the consistent performance of the platforms. Because many researchers have concentrated on proving the efficacy of new formulations, from now on, researchers should focus their work on establishing safety and stability.

Herein, we have gathered recent articles about the chitosan-based multifunctional platform regarding the purpose of its use. Chitosan may be one of the well-explored and widely used polymers as a platform, yet there is more to be studied. We hope that this review will give an insight into designing better chitosan platforms and ultimately, further help to extend the boundaries of the use of chitosan-based multifunctional platforms.

## Figures and Tables

**Table 1:** Various therapeutic uses and applications of chitosan-based platforms.

Therapeutic Use	Application	Key Findings	Reference
Embolization	Deformable chitosan microspheres	•Highly spherical and porous chitosan microspheres were formed •Deformable microspheres were able to pass through the microcatheter.	[25]
	Adriamycin-loaded alginate-chitosan microcapsule	•Drug rapidly released in acidic condition. •Successful occlusion of rabbit renal artery. •Observed synergistic effect of embolization and anti-cancer drug.	[26]
	Doxorubicin (DX)-loaded chitosan microsphere	•Microspheres were designed and evaluated under different conditions. •Observed synergistic effect of embolization and anti-cancer drug.	[27]
	Superparamagnetic iron oxides (SPIOs) loaded chitosan microsphere	•Deformable microspheres were able to pass through the microcatheter. •The released SPIOs from the microsphere were detectable via magnetic resonance imaging (MRI) allowing to monitor the embolization outcome of the patient.	[28]
Theragnosis	DX-loaded ZnO folate-chitosan quantum dot	•Folate allows receptor-specific targeting of the anticancer drug. •Long-term fluorescence stability of ZnO allows in vivo visualization.	[29]
	Cy5.5-labled paclitaxel-loaded chitosan nanoparticle	•The anti-cancer drug was selectively delivered to tumor tissue by enhanced permeation and retention effect. •The Cy5.5 dye in the tumor tissue was detectable by near-infrared fluorescence detection.	[30]
	Chitosan-based DX-loaded magnetic nanoparticle	•The DX and nanoparticles were released in a pH-dependent manner. •Under acidic condition, the tumor tissue was detectable by MRI.	[31]
Tissue engineering	Chitosan hyaluronic acid (HA) hydrogel	•Addition of HA showed tighter networks, smaller pore size, increased stability. •HA provides a suitable environment for chondrocytes culture.	[32]
	Alginate-*O*-carboxymethyl chitosan hydrogel	•The modified chitosan was able to enhance the adhesion, differentiation and survival of adipose-derived stem cells on the scaffold.	[33]
	Arginine-glycine-aspartate (RGD)-conjugated chitosan scaffold	•Applied specific method to fabricate the RGD-conjugated, crosslinked chitosan scaffold. •Mesenchymal stem cells were well adhered, differentiated and survived on the scaffold.	[34]
Wound healing	Chitosan-pectin-TiO_2_ nanodressing	•Addition of titanium dioxide improved mechanical strength. •The nanodressing showed good anti-microbial and blood-compatibility along with significant wound healing and closure rate.	[35]
	Human epidermal growth factor (EGF)-loaded chitosan film	•EGF showed significant vascular healing effect compared to conventional formulation.	[36]
	Neurotensin (NT)-loaded chitosan dressing	•Bioactive NT enhanced the healing effect on diabetic wounds. •Chitosan dressing was able to modulate immune response along with sustained release.	[37]
